# Identification of a promiscuous conserved CTL epitope within the SARS-CoV-2 spike protein

**DOI:** 10.1080/22221751.2022.2043727

**Published:** 2022-03-01

**Authors:** Sheng Jiang, Shuting Wu, Gan Zhao, Yue He, Xinrong Guo, Zhiyu Zhang, Jiawang Hou, Yuan Ding, Alex Cheng, Bin Wang

**Affiliations:** a Key Laboratory of Medical Molecular Virology (MOE/NHC/CAMS), School of Basic Medical Sciences, Shanghai Medical College (SHMC), Fudan University, Shanghai, People’s Republic of China; b National Clinical Research Center for Aging and Medicine, Huashan Hospital, Fudan University, Shanghai, People’s Republic of China; c Advaccine Biopharmaceutics (Suzhou) Co. LTD, Suzhou, Jiangsu Province, People’s Republic of China; d Colby College, Waterville, ME, USA

**Keywords:** SARS-CoV-2, Spike; MHC-I epitope, cellular immunity; HLA alleles

## Abstract

The COVID-19 disease caused by infection with SARS-CoV-2 and its variants is devastating to the global public health and economy. To date, over a hundred COVID-19 vaccines are known to be under development, and the few that have been approved to fight the disease are using the spike protein as the primary target antigen. Although virus-neutralizing epitopes are mainly located within the RBD of the spike protein, the presence of T cell epitopes, particularly the CTL epitopes that are likely to be needed for killing infected cells, has received comparatively little attention. This study predicted several potential T cell epitopes with web-based analytic tools and narrowed them down from several potential MHC-I and MHC-II epitopes by ELIspot and cytolytic assays to a conserved MHC-I epitope. The epitope is highly conserved in current viral variants and compatible with a presentation by most HLA alleles worldwide. In conclusion, we identified a CTL epitope suitable for evaluating the CD8+ T cell-mediated cellular response and potentially for addition into future COVID-19 vaccine candidates to maximize CTL responses against SARS-CoV-2.

## Introduction

Severe Acute Respiratory Syndrome Coronavirus 2 (SARS-CoV-2) was first identified in Wuhan at the end of 2019, spread at unprecedented speed, and became a disaster to human beings worldwide [1]. Effective vaccines, antiviral drugs, and treatments have high priorities to defend against such challenges. SARS-CoV-2 has four main structural proteins: the envelope, membrane, nucleocapsid, and spike protein considered for inclusion in vaccines. The spike protein has a receptor-binding domain (RBD) that specifically binds to human angiotensin-converting enzyme 2 (hACE2) as a receptor and mediates virus entry into the host cell [2,3]. Neutralizing antibodies recognizing the RBD can block the spike protein from binding to the hACE2 and inhibit virus entry [4,5]. Therefore, spike protein has been the primary choice as the immunogen in candidate vaccines.

Although protection against disease via vaccine-induced neutralizing antibodies has been demonstrated, the elimination of SARS-CoV-2 infection within the host is also essential. The numbers of mild and asymptomatic cases have been rising dramatically in recent years, and such cases remain infective, prolonging viral dissemination [6]. To eliminate the viral infection, induction of a potent antigen-specific CD8+ T cell response by vaccination is probably critical [7,8]. T cell immunity is indispensable for viral clearance, as demonstrated in animal models infected with viruses like JEV, DENV, and recently Zika, among others [7,9,10]. Few of the currently available methods can monitor virus-specific CD8+ T cells, and consequently, few studies have investigated whether virus-specific CTLs influence the pathology of COVID-19 or contribute to the elimination of the virus. Identification of peptides recognized by CTLs would help address these issues by enabling analysis of the distribution, function, and phenotype of specific CD8+ T cells in SARS-CoV-2-infected mice and facilitating studies of the T effect cell immune response on virus clearance in such models [11–13].

To activate a viral-specific CD8+ T cell response, the vaccine must contain highly active major histocompatibility complex class I (MHC-I) epitopes that MHC-I molecules can present to interact with CD8+ T cell receptors (TCR). The potentiation of viral-specific CD8+ T cell responses depends on the high affinity and avidity of MHC-I and TCR binding. There is a lack of information on the CD8+ T cell-recognized epitopes within the spike antigen; consequently, only overlapping peptide pools covering the whole region of spike antigen have been used routinely to evaluate cell-mediated immunity (CMI) of vaccine candidates [14–16]. A few reports have suggested that CTL epitopes are present within the spike protein, but only one epitope has been reported among the potential sequences discovered [17]. Identifying those CD8+ T cell epitopes would provide an important tool to evaluate the T cell immunity in vaccinated individuals or patients and was undertaken here.

This study utilized web-based tools to analyze the potentials for transportation associated with antigen processing (TAP) in the human MHC-I epitopes that were predicted by the Immune Epitope Database analysis (IEDB) resource [18] to be present in peptide pools covering the N-terminal domain (NTD) and receptor-binding domain (RBD) of the spike protein. We demonstrated that peptide 2 (YYVGYLQPRTFLLKY), although it did not give the highest score in the web-based analysis of immunogenicity, was the best epitope for inducing a robust antigen-specific IFN-γ producing CD8+ T response as defined by ELIspot assay. This epitope sequence is also highly conserved among currently discovered SARS-CoV-2 variants.

## Materials and methods

### Mice

Female Balb/c mice (6-8 weeks of age) were purchased from Beijing Vital Laboratory Animal Technology Co., Ltd. (Beijing, China) and Shanghai Jiesjie Laboratory Animal Co., Ltd. (Shanghai, China), and were kept in SPF conditions. All animal experiments were approved by the Experimental Animals Committee of SHMC, and all methods were carried out in accordance with relevant guidelines and regulations. This study was carried out in compliance with the ARRIVE guidelines. After testing, all mice were sacrificed by euthanasia with isoflurane treatment.

### Peptide pool derived from SARS-CoV-2 spike protein

The spike receptor-binding domain (RBD) peptide pool (SARS-CoV-2 spike protein aa258-518) published previously [15] was used for the study (Table 1), which was pool 2 in our previous study and renamed as pool 1 in this study. The peptide pool 5 covered the spike S2 region (SARS-CoV-2 spike protein aa1015-1275) in our previous study was renamed as pool 2 in this study. The peptides (Table 1 & sTable 2) were synthesized by Genescript (Nanjing, China).

**Table 1.** Overlapping Peptide Pool 1.

Peptide number	Sequence	Start	End	aa
1	TAGAAAYYVGYLQPR	258	272	15
2	YYVGYLQPRTFLLKY	264	278	15
3	QPRTFLLKYNENGTI	270	284	15
4	LKYNENGTITDAVDC	276	290	15
5	GTITDAVDCALDPLS	282	296	15
6	VDCALDPLSETKCTL	288	302	15
7	PLSETKCTLKSFTVE	294	308	15
8	CTLKSFTVEKGIYQT	300	314	15
9	TVEKGIYQTSNFRVQ	306	320	15
10	YQTSNFRVQPTESIV	312	326	15
11	RVQPTESIVRFPNIT	318	332	15
12	SIVRFPNITNLCPFG	324	338	15
13	NITNLCPFGEVFNAT	330	344	15
14	PFGEVFNATRFASVY	336	350	15
15	NATRFASVYAWNRKR	342	356	15
16	SVYAWNRKRISNCVA	348	362	15
17	RKRISNCVADYSVLY	354	368	15
18	CVADYSVLYNSASFS	360	374	15
19	VLYNSASFSTFKCYG	366	380	15
20	SFSTFKCYGVSPTKL	372	386	15
21	CYGVSPTKLNDLCFT	378	392	15
22	TKLNDLCFTNVYADS	384	398	15
23	CFTNVYADSFVIRGD	390	404	15
24	ADSFVIRGDEVRQIA	396	410	15
25	RGDEVRQIAPGQTGK	402	416	15
26	QIAPGQTGKIADYNY	408	422	15
27	TGKIADYNYKLPDDF	414	428	15
28	YNYKLPDDFTGCVIA	420	434	15
29	DDFTGCVIAWNSNNL	426	440	15
30	VIAWNSNNLDSKVGG	432	446	15
31	NNLDSKVGGNYNYLY	438	452	15
32	VGGNYNYLYRLFRKS	444	458	15
33	YLYRLFRKSNLKPFE	450	464	15
34	RKSNLKPFERDISTE	456	470	15
35	PFERDISTEIYQAGS	462	476	15
36	STEIYQAGSTPCNGV	468	482	15
37	AGSTPCNGVEGFNCY	474	488	15
38	NGVEGFNCYFPLQSY	480	494	15
39	NCYFPLQSYGFQPTN	486	500	15
40	QSYGFQPTNGVGYQP	492	506	15
41	PTNGVGYQPYRVVVL	498	512	15

Notes:

1. The peptides covered the entire sequence of 258–512 amino acids in the spike protein as previously named as the Pool 2 [15];

2. The peptides were synthesized with an average length of 15 amino acids and nine amino acids overlapping each other.

### Immunization

The mice were injected twice with a two-week interval via the intramuscular route (i.m.) with 25 μg of pVAX-S-WT, made from the wild-type sequence of the full-length spike protein of the SARS-CoV-2 (SARS-CoV-2/WH-09/human/2020/CHN), or with pGX9501 expressing a synthetic, optimized sequence of the SARS-CoV-2 full-length spike glycoprotein [15]. Electroporation was applied with the Cellectro2000™ device. Serum samples and spleens were collected 14 days after the second immunization.

### IEDB analysis for SARS-CoV-2 MHC-I epitope identification

An explorative panel of SARS-CoV-2-derived epitopes with the highest predicted affinity to MHC Class I molecules was defined by Immune Epitope Database analysis (www.IEDB.org). The selection was based on internal predictions using NetMHCpan Version EL4.1. All predicted epitopes with a percentile rank of < 2 were selected for a further MHC-I processing analysis using MHC-NP methods. Simultaneously, those epitopes were analyzed in MHC-I immunogenicity to check if the peptide sequence was consistent with this allele's site preference. After applying the above three analysis methods, peptides with a percentile rank of < 0.5, a TAP total score of > −1, and an immunogenicity score of > 0 were subjected to an ELIspot assay to evaluate their ability to elicit a T cell IFN-γ response.

### Cytotoxic lymphocyte (CTL) killing ability

A single-cell suspension of splenocytes from naïve syngeneic mice was diluted to 1.5 × 10^8^/ml in RPMI1640 containing 10% FBS and 2% penicillin and streptomycin pulsed at 37°C with or without 5 μg/ml peptides as described previously [19]. After 4 h, eflour450 (eBioscience, 65-0842-85) at 5 mM (high concentration) was used to label peptide-pulsed cells at room temperature in the dark. Non-peptide-pulsed cells were labelled with a low concentration of eflour450 at 0.5 mM. After being rinsed three times with PBS, 4 × 10^6^ labelled and peptide-pulsed cells and an equal number of labelled non-peptide-pulsed cells were adoptively transferred by tail vein injections into mice that had previously been immunized. Six hours later, the percentage of labelled cells in spleens was detected with LSRFortessa flow cytometry (BD) and analyzed by FlowJo (TreeStar). The following formula calculated the specific cell lysis: Specific cell lysis ability% = (1-(percentage of cells incubated with peptide/percentage of cells incubated without peptide)) x100%.

### IFN-γ ELIspot

Splenocytes were collected from individual mice into RPMI1640 media supplemented with 10% FBS (R10, Gibco) and penicillin/streptomycin and processed into single-cell suspensions. ELIspot assays were performed using Mouse IFN-γ ELIspot plates (Dakewei Biotech Co., Ltd, 2210006, Shenzhen, China). The ELIspot plates were washed 5 times at RT with 100 μL of PBS per well then incubated with 200 μL of R10 for 10 min before the cells were plated. Two hundred fifty thousand mouse splenocytes, CD4+, or CD8+ T cells were plated into each well and stimulated for 16 h with 15-mer peptides from the SARS CoV-2 spike peptide pools that overlapped by nine amino acids as previously described [15]. Each peptide was at a final concentration of 1 μg in 100 μl R10 per well. The spots were developed based on the manufacturer's instructions. R10 and cell stimulation cocktails (Invitrogen) were used for negative and positive controls. Spots were scanned and quantified by AID ELIspot READER (AID, Germany). After subtracting the negative control wells, spot-forming units (SFU) per million cells were calculated.

### Statistical analysis

The statistical analysis methods and sample sizes (n) are specified in the results section or figure legends for all quantitative data. All values are reported as means ± sem with the indicated sample size. No samples were excluded from the analysis. All relevant statistical tests were two-sided and *p* values less than 0.05 were considered statistically significant. All animal studies were performed with randomized animal selection. Statistics were performed using GraphPad Prism 7 software. In all data, * *p* < 0.05, ** *p* < 0.01, *** *p* < 0.001, and **** *p* < 0.0001.

## Results

### Strong CD8+ CTL epitope activity is embedded in an overlapping peptide pool 1 that covers the NTD and RBD region of the spike protein

**Figure 1.**
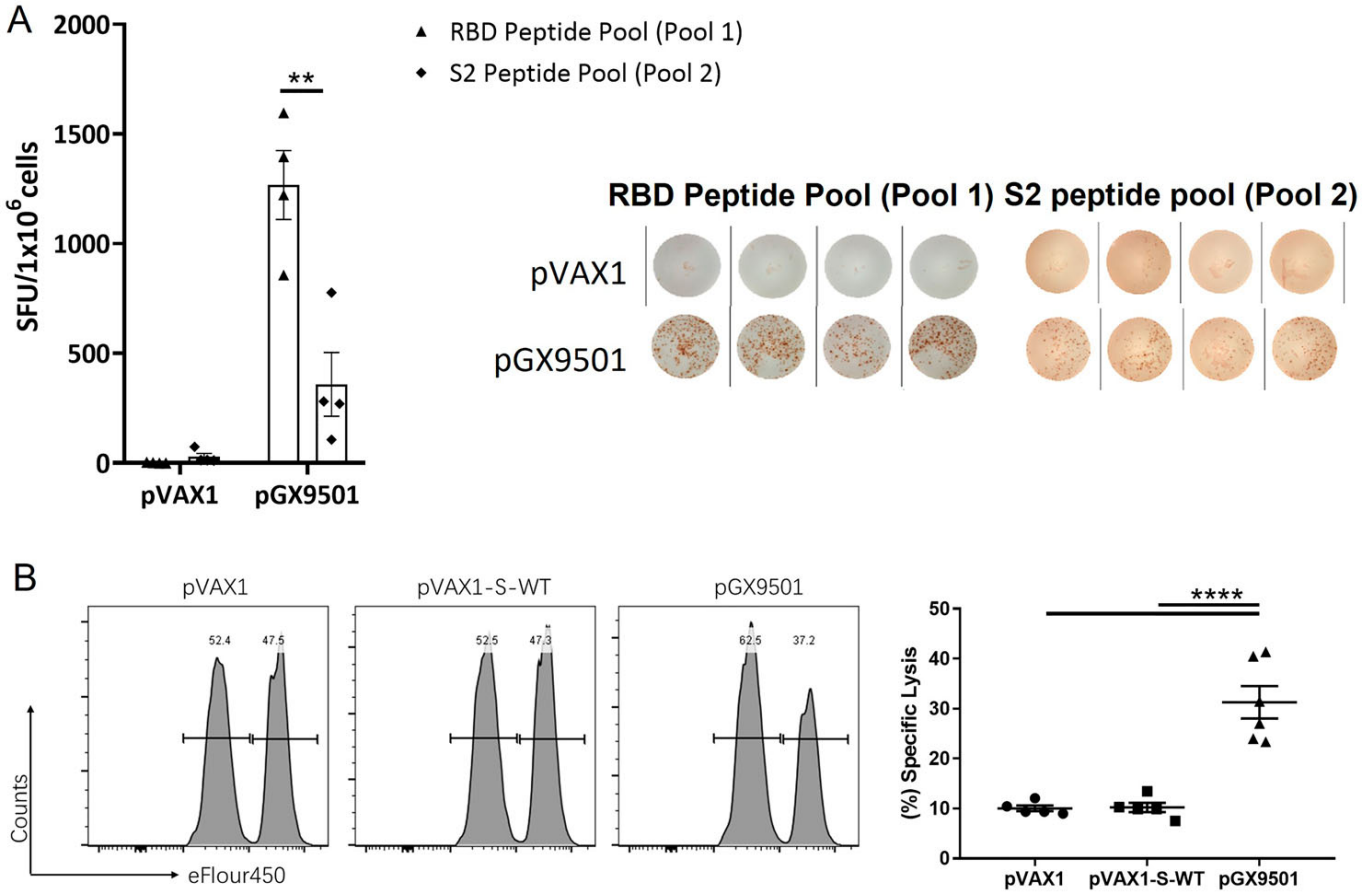
**Peptide pool 1 induced strong T cell responses in Balb/c mice.** Balb/c mice (n = 5/group) were immunized twice two weeks apart with 25 μg pGX9501 or pVAX1 (empty vector). T cell responses were analyzed on day 14 after the second injection. (A) Splenocytes were harvested, and IFN-γ ELIspot T cell responses were measured after stimulation for 20 h with overlapping peptide pools 1 or 2. (B) Antigen-specific cytotoxic lymphocyte (CTL) killing activity was evaluated by an in vivo CTL assay. Target cells at 4 × 10^6^/ml from naïve mice were peptide-pulsed with pool 1 then labelled with a high concentration of eFlour450 in vitro. Control cells were non-peptide-pulsed cells and labelled with a low concentration of eFluor450. The cells were mixed and transferred i.v. into immunized mice. After 5 h, splenocytes were harvested, and the intensity of eFlour450 peptide labelled target cells was compared with the non-peptide-labelled negative control cells by flow cytometry. pVAX1-s-WT was made from the wild type sequence of the full-length spike protein of the SARS-CoV-2(SARS-CoV-2/WH-09/human/2020/CHN) was subcloned into the pVAX1. The sequence of the same region was optimized via SynCon technology, synthesized, and cloned into pVAX1 as the pGX9501.

When Balb/c mice were immunized twice with the pGX9501 DNA vaccine expressing the spike protein of SARS-CoV-2, a higher level of IFN-γ expression by splenocytes was more often seen by the ELIspot assay when the cells were stimulated in vitro with spike peptide pool 1 (Table 1) compared with pool 2 (sTable 2 & Figure 1A). In addition, when an in vivo CTL assay was done with identically immunized animals, the same peptide pool 1 gave a strong CTL response in vivo (Figure 1B), suggesting that MHC-I epitope(s) were present within pool 1.

### Screening and identification of an MHC-I epitope in peptide pool 1.

**Figure 2.**
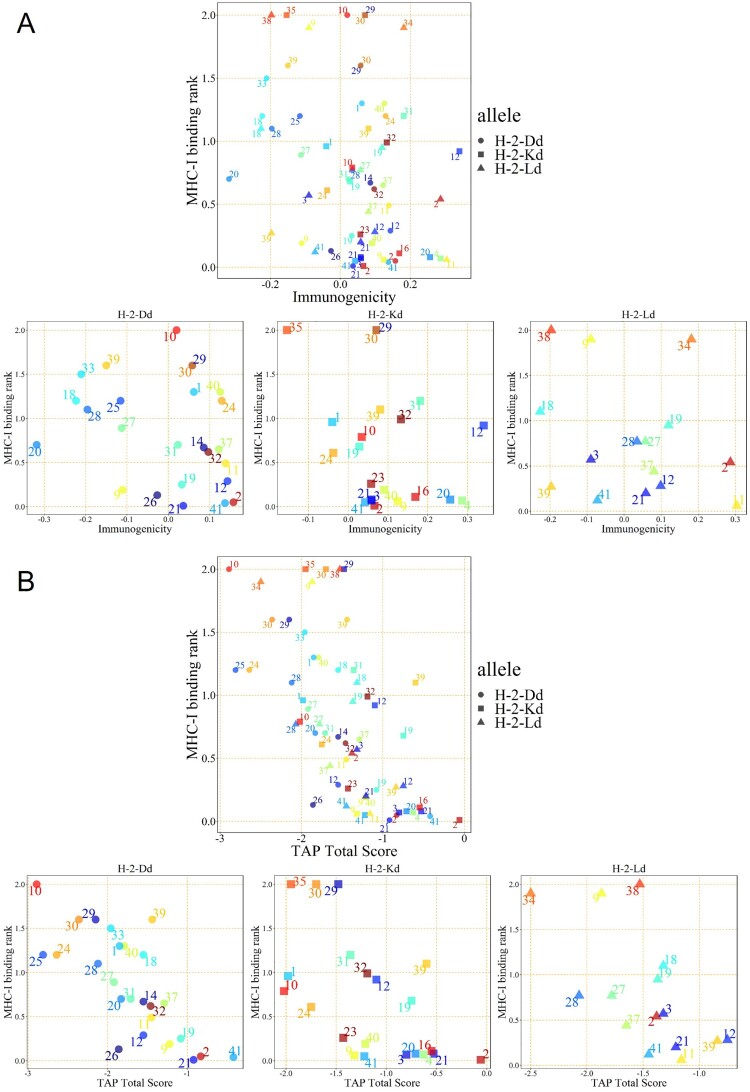
**IEDB prediction scores of peptides in pool 1.** CTL epitope peptides were screened by integrating MHC-I binding prediction, MHC-I immunogenicity (A), and MHC-NP (B) prediction from three H-2d MHC-I alleles. The numbers on the graph are the peptide identification numbers. The different colours are corresponding to each peptide ID.

**Figure 3.**
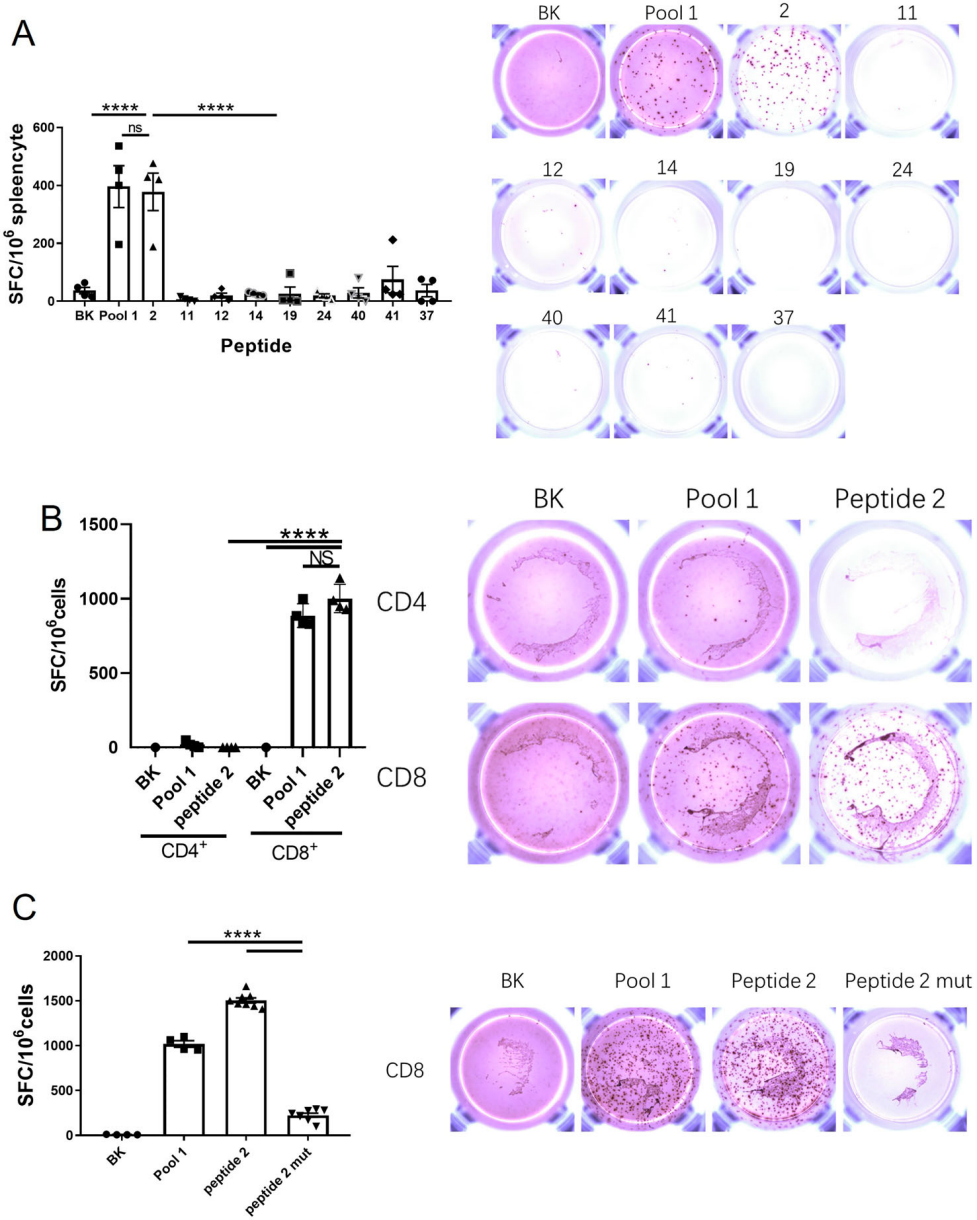
**Peptide 2 is identified as a CD8+ CTL epitope.** Balb/c mice were immunized with the pGX9501. (A) Splenocytes were obtained and used to analyze antigen-specific T-cell induction in the IFN-γ ELIspot assay using in vitro stimulation with the indicated peptides (the peptide with an MHC-I binding RANK < 2 and showing the highest TAP total score or Immunogenicity score in different alleles) was selected for the IFN-γ ELIspot assay. (B) CD4+ T cells and (C) CD8+ T cells were sorted, and specific T-cell induction of IFN-γ-secretion was assayed.

To seek T cell-relevant epitopes, we placed the entire 41 peptide sequences from peptide Pool 1 into the Immune Epitope Database analysis (IEDB, http://www.iedb.org/). An evaluation method was established by integrating MHC-I binding prediction, MHC-I immunogenicity, and MHC natural processing (MHC-NP) prediction from three H-2d MHC-I alleles to improve prediction results (Table 2). In the H-2D^d^ allele, Peptide 2 showed good MHC-I binding ability, immunogenicity, and TAP ability. In the H-2K^d^ allele, Peptide 12 showed the strongest immunogenicity, and Peptide 2 presented the most potent MHC-I binding ability and TAP ability among all peptides. In the H-2L^d^ allele, both Peptide 2 and Peptide 11 showed the strongest immunogenicity, while Peptide 12 emerged as having the most potent TAP ability (Figure 2A & B). Consequently, The Peptides (2, 11, 12, and 41) for which the MHC-I binding RANK was < 2 and showed the greatest TAP total score or Immunogenicity score in the various alleles were selected for the IFN-γ ELIspot assay. As shown in Figure 3A, Peptide 2 from Pool 1 presented the best stimulation to induce the IFN-γ secretion compared to the other two selected peptides. Thus, Peptide 2, consisting of 15 amino acids, stimulated CD8^+^ T cells via MHC-I or/and CD4^+^ T cells via MHC-II. To identify which T cell type was stimulated by Peptide 2, purified CD4^+^ T cells or CD8^+^ T cells were used (sFigure 1). Peptide 2 stimulated CD8^+^ T cells but not CD4^+^ cells, indicating that it can only be presented by MHC-I (Figure 3B). To further investigate its sequence specificity, we mutated several predicted anchor amino acids of Peptide 2 according to the preferences of the H-2d MHC-I allele [20–23]. The mutated Peptide 2 had a low MHC-I binding score in the IEDB prediction (sTable 3) and showed a significantly reduced ability to stimulate IFN-γ secretion by CD8^+^ T cells (Figure 3C). Furthermore, we compared this peptide with the MHC-I peptides reported in a previous study [17] (e.g. S526-533, GPKKSTNL) and found that Peptide 2 was significantly more potent in the induction of IFN-γ secreting T cells than the previously reported peptides (sFigure 2).

**Table 2.** MHC-I epitope analysis for Overlapping Peptide Pool 1.

Allele	peptide number	MHC-I binding	immunogenicity	Proteasome Score	TAP Score	MHC Score	Processing Score	TAP Total Score
H-2-Dd	21	0.01	0.03612	1.44	1.12	−3.49	2.57	−0.92
H-2-Dd	41	0.04	0.13706	1.74	0.4	−2.56	2.14	−0.42
H-2-Dd	2	0.05	0.1573	1.36	1.07	−3.26	2.43	−0.83
H-2-Dd	26	0.13	−0.02676	1.38	1.27	−4.51	2.65	−1.86
H-2-Dd	9	0.19	−0.11058	1.27	0.99	−3.48	2.26	−1.22
H-2-Dd	19	0.25	0.03263	1.33	1.04	−3.45	2.37	−1.08
H-2-Dd	12	0.29	0.1431	1.05	1.29	−3.9	2.35	−1.55
H-2-Dd	11	0.49	0.1386	1.36	1.21	−4.02	2.57	−1.45
H-2-Dd	32	0.62	0.0966	1.19	1.17	−3.83	2.36	−1.46
H-2-Dd	37	0.65	0.12191	1.38	1.09	−3.76	2.47	−1.29
H-2-Dd	14	0.67	0.08562	1.39	0.82	−3.76	2.21	−1.55
H-2-Dd	31	0.7	0.023	1.31	1.15	−4.17	2.46	−1.71
H-2-Dd	20	0.7	−0.31841	1.47	1.3	−4.6	2.77	−1.83
H-2-Dd	27	0.89	−0.11289	1.4	1.21	−4.54	2.61	−1.92
H-2-Dd	28	1.1	−0.19576	0.98	1.12	−4.22	2.1	−2.12
H-2-Dd	24	1.2	0.12947	1.27	0.71	−4.63	1.98	−2.64
H-2-Dd	25	1.2	−0.11559	1.43	0.15	−4.39	1.58	−2.81
H-2-Dd	18	1.2	−0.22309	1.33	1.17	−4.05	2.5	−1.55
H-2-Dd	40	1.3	0.1256	1.38	1.34	−4.5	2.71	−1.79
H-2-Dd	1	1.3	0.06158	1.24	1.23	−4.32	2.47	−1.85
H-2-Dd	33	1.5	−0.21085	1.03	1.24	−4.23	2.27	−1.96
H-2-Dd	29	1.6	0.05792	1.52	0.5	−4.17	2.02	−2.15
H-2-Dd	30	1.6	0.05792	1.35	0.46	−4.17	1.8	−2.36
H-2-Dd	39	1.6	−0.15021	1.25	1.15	−3.84	2.41	−1.44
H-2-Dd	10	2	0.01977	1.1	0.24	−4.23	1.34	−2.89
H-2-Kd	2	0.01	0.06572	1.24	0.48	−1.78	1.72	−0.06
H-2-Kd	41	0.05	0.04196	1.74	0.46	−3.43	2.2	−1.22
H-2-Kd	9	0.06	0.12441	1	0.23	−2.55	1.23	−1.32
H-2-Kd	4	0.07	0.28634	1.34	0.37	−2.34	1.71	−0.63
H-2-Kd	3	0.07	0.05892	1.16	0.37	−2.34	1.54	−0.8
H-2-Kd	20	0.08	0.25644	1.45	0.49	−2.65	1.93	−0.71
H-2-Kd	21	0.08	0.05832	1.75	0.36	−2.65	2.12	−0.53
H-2-Kd	16	0.11	0.16858	1.36	0.4	−2.32	1.77	−0.55
H-2-Kd	40	0.19	0.0905	1.38	1.32	−3.9	2.69	−1.21
H-2-Kd	23	0.26	0.0573	1.31	0.44	−3.18	1.75	−1.43
H-2-Kd	24	0.61	−0.0378	1.07	0.2	−3.02	1.27	−1.75
H-2-Kd	19	0.68	0.0279	1.33	1.2	−3.27	2.52	−0.75
H-2-Kd	10	0.79	0.034	1.1	0.23	−3.35	1.33	−2.02
H-2-Kd	12	0.92	0.34063	1.45	0.59	−3.13	2.03	−1.1
H-2-Kd	1	0.96	−0.04018	1.24	1.29	−4.51	2.53	−1.98
H-2-Kd	32	0.99	0.13255	1.19	1.18	−3.56	2.37	−1.19
H-2-Kd	39	1.1	0.0801	1.25	1.31	−3.16	2.56	−0.6
H-2-Kd	31	1.2	0.1811	1.48	0.48	−3.32	1.96	−1.36
H-2-Kd	29	2	0.07062	1.52	0.5	−3.5	2.02	−1.48
H-2-Kd	30	2	0.07062	1.35	0.46	−3.5	1.8	−1.7
H-2-Kd	35	2	−0.15381	1.42	1.16	−4.52	2.57	−1.95
H-2-Ld	11	0.06	0.30371	1.36	0.98	−3.5	2.34	−1.16
H-2-Ld	41	0.12	−0.07228	1.74	0.35	−3.54	2.09	−1.45
H-2-Ld	21	0.2	0.05832	1.44	0.99	−3.65	2.44	−1.21
H-2-Ld	39	0.27	−0.19696	1.25	0.91	−3	2.16	−0.84
H-2-Ld	12	0.28	0.09851	1.05	0.94	−2.75	2	−0.75
H-2-Ld	37	0.44	0.0801	1.38	0.99	−4.01	2.37	−1.65
H-2-Ld	2	0.54	0.28634	1.41	1.19	−3.97	2.6	−1.38
H-2-Ld	3	0.57	−0.08994	1.52	1.13	−3.97	2.65	−1.32
H-2-Ld	27	0.77	0.0573	1.4	1.21	−4.39	2.61	−1.78
H-2-Ld	28	0.77	0.03448	0.98	1.12	−4.18	2.1	−2.07
H-2-Ld	19	0.95	0.11915	1.53	1.42	−4.32	2.95	−1.37
H-2-Ld	18	1.1	−0.22669	1.33	1.17	−3.82	2.5	−1.32
H-2-Ld	34	1.9	0.1811	0.91	1.16	−4.58	2.07	−2.5
H-2-Ld	9	1.9	−0.08994	1.27	0.99	−4.13	2.26	−1.87
H-2-Ld	38	2	−0.19696	0.99	0.28	−2.8	1.27	−1.53

Notes:

1. MHC-I binding score was between 0 and 2. < 0.5 strong binder, 0.5-2 weak binder, > 2 non-binder.

2. A high Immunogenicity score indicates that the degree of the peptide conformity to sequence preference was good.

3. The higher the TAP total score, the higher the likelihood that the peptide will be presented after being swallowed by DCs.

### Analysis of Peptide 2 epitope conservation and HLA distribution

**Figure 4.**
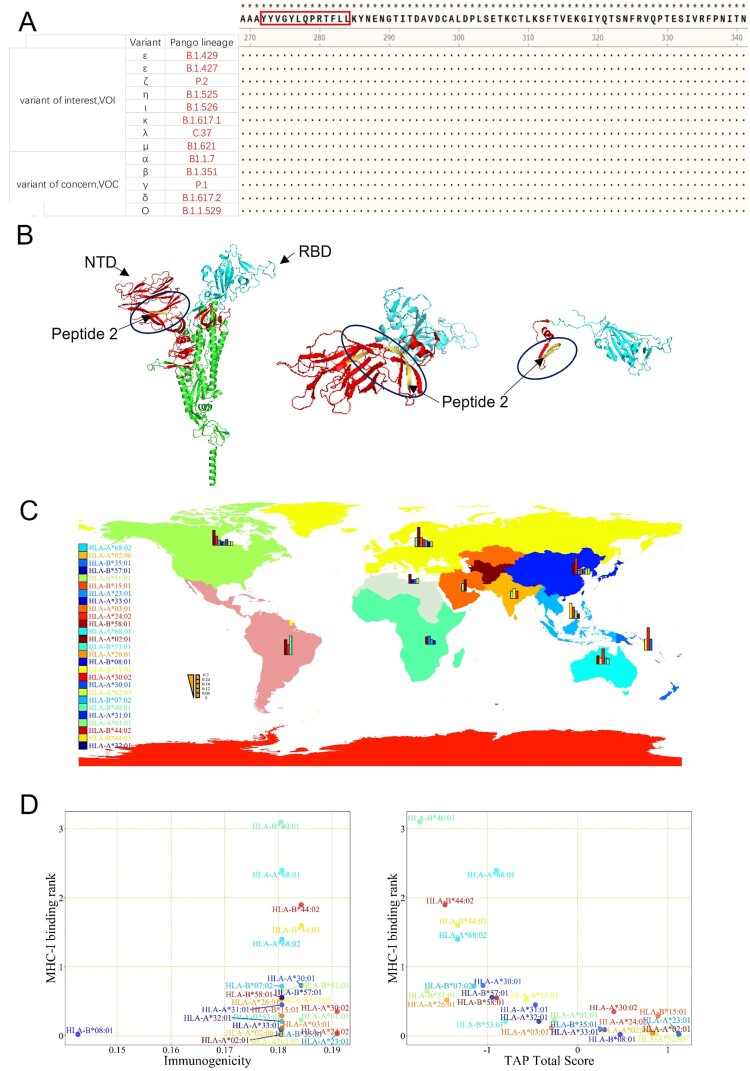
**Conserved Sequence & MHC-I HLA Analysis of Peptide 2.** (A) The sequence of Peptide 2 was highly conserved in the 11 virus variants that have been identified as the variants of interest and the variants of concern, as published by WHO. (B) Position of Peptide 2 (yellow marked segment) in the stereoscopic structure of the spike protein. (C) The global distribution of HLA alleles. (D) Analysis of Peptide 2 by integration of MHC-I binding prediction, MHC-I immunogenicity, and MHC-NP prediction from the HLA alleles.

We compared the sequence of Peptide 2, YYVGYLQPRTFLLKY (amino acid 264-278), with the sequences in the current SARS-CoV-2 variants-of-concern (VOC) and variants-of-interest (VOI) posted by WHO, including the latest Omicron variant. We observed that this sequence is highly conserved among those variants (Figure 4A) and located at the end of the NTD of the Spike protein and upstream of the RBD (Figure 4B). Hence, this highly conserved epitopic sequence provides a valuable tool for evaluating the CD8^+^ T cell-mediated responses to vaccine evaluation both in animals and humans.

Since MHC-I-biased expression patterns in different populations are globally diversified and variable, a peptide sequence that can be recognized by one population may not be recognized by others. To investigate if this is the case with Peptide 2, we performed an HLA allele analysis for different regions to assess binding to one or more of the 27 prevalent MHC-I molecules, including HLA-A (01:01, 02:01, 02:03, 02:06, 03:01, 11:01, 23:01, 24:02, 26:01, 30:01, 30:02, 31:01, 32:01, 33:01, 68:01, 68:02), and HLA-B (07:02, 08:01, 15:01, 35:01, 40:01, 44:02, 44:03, 51:01, 53:01, 57:01, 58:01), as shown in Figure 4C & Table 3. The frequency by HLAs was calculated with the online analysis tool at http://www.allelefrequencies.net/. We also evaluated the MHC-I binding ability, immunogenicity, and TAP potential of Peptide 2 on different HLA alleles by IEDB (Figure 4D & Table 4). We set the MHC-I binding score when it was < 0.2, and then the immunogenicity was >0 as a basis for the determination. The results indicated that Peptide 2 could be recognizable by the HLA-A*02:01 allele (most in Europe and America), HLA-B*08:01 allele (in Europe and Australia), HLA-A*23:01 allele (in North Africa and Sub-Saharan Africa), HLA-A*02:03 allele (in Southeast Asia), HLA-A*24:02 allele (in Oceania), HLA-A*02:06 allele (in North America, North-East Asia, and Oceania), HLA-A*33:01 allele (in China and Pakistan), HLA-B*35:01 allele (in Oceania), and HLA-A*03:01 allele (in Europe). These findings suggest that Peptide 2 could be well recognized by the most frequent HLA alleles of the worldwide population and can therefore be considered to be promiscuous.

**Table 3.** Geographic Distribution of HLA allele.

Continent	Allele	Frequency	Allele	Frequency
Australia	HLA-A*24:02	0.2	HLA-B*07:02	0.08
	HLA-A*02:01	0.11	HLA-B*40:01	0.07
	HLA-A*11:01	0.08		
Europe	HLA-A*02:01	0.26	HLA-B*07:02	0.08
	HLA-A*01:01	0.12	HLA-B*08:01	0.07
	HLA-A*03:01	0.12	HLA-B*51:01	0.07
	HLA-A*24:02	0.1		
North Africa	HLA-A*02:01	0.12	HLA-B*51:01	0.07
			HLA-B*08:01	0.05
			HLA-B*35:01	0.05
North America	HLA-A*02:01	0.2	HLA-B*35:01	0.08
	HLA-A*24:02	0.12	HLA-B*07:02	0.07
			HLA-B*08:01	0.05
			HLA-B*15:01	0.05
			HLA-B*44:03	0.05
			HLA-B*51:01	0.05
North-East Asia	HLA-A*24:02	0.22	HLA-B*51:01	0.08
	HLA-A*02:01	0.14	HLA-B*35:01	0.07
			HLA-B*15:01	0.07
			HLA-B*44:03	0.06
			HLA-B*07:02	0.05
Oceania	HLA-A*24:02	0.3	HLA-B*35:01	0.15
	HLA-A*11:01	0.15		
South and Central America	HLA-A*02:01	0.2	HLA-B*40:01	0.25
	HLA-A*24:02	0.14		
South Asia	HLA-A*11:01	0.13		
	HLA-A*01:01	0.1		
	HLA-A*24:02	0.1		
South-East Asia	HLA-A*11:01	0.2	HLA-B*40:01	0.1
	HLA-A*24:02	0.15	HLA-B*58:01	0.06
Sub-Saharan Africa	HLA-A*23:01	0.11	HLA-B*07:02	0.06
	HLA-A*02:01	0.1	HLA-B*08:01	0.05
Western Asia	HLA-A*02:01	0.15		
	HLA-A*01:01	0.1		

Notes:

1. Frequency means the proportion of HLA allele present in the survey population. This table shows the allele frequencies that were equal to higher than 0.05 in the continent.

**Table 4.** Human MHC-I epitope analysis for peptide 2.

Allele	Peptide number	MHC-I binding	Immunogenicity	Proteasome Score	TAP Score	MHC Score	Processing Score	TAP Total Score
HLA-A*02:01	2	0.02	0.18048	1.39	0.39	−0.66	1.78	1.12
HLA-B*08:01	2	0.02	0.14268	1.39	0.39	−1.31	1.78	0.47
HLA-A*23:01	2	0.03	0.19092	1.36	1.13	−1.38	2.5	1.12
HLA-A*02:03	2	0.03	0.18064	1.39	0.39	−0.91	1.78	0.87
HLA-A*24:02	2	0.04	0.19092	1.36	1.13	−1.67	2.5	0.83
HLA-A*02:06	2	0.04	0.18064	1.39	0.39	−0.96	1.78	0.82
HLA-A*33:01	2	0.09	0.18048	0.76	0.68	−1.13	1.43	0.3
HLA-B*35:01	2	0.1	0.18064	1.41	1.19	−2.35	2.6	0.25
HLA-A*03:01	2	0.12	0.18064	0.89	0.18	−1.35	1.06	−0.29
HLA-A*32:01	2	0.21	0.18064	1.39	0.39	−2.21	1.78	−0.43
HLA-B*53:01	2	0.21	0.18064	1.41	1.19	−3.4	2.6	−0.8
HLA-A*01:01	2	0.23	0.18424	1.41	1.24	−2.91	2.65	−0.26
HLA-B*15:01	2	0.29	0.18064	1.41	1.28	−1.79	2.69	0.89
HLA-A*30:02	2	0.35	0.19092	1.41	1.28	−2.29	2.69	0.4
HLA-A*31:01	2	0.45	0.18064	0.76	0.68	−1.9	1.43	−0.47
HLA-A*26:01	2	0.52	0.18048	1.41	1.28	−4.14	2.69	−1.45
HLA-A*11:01	2	0.53	0.18064	0.89	0.18	−1.63	1.06	−0.57
HLA-B*58:01	2	0.55	0.18064	1.36	1.06	−3.33	2.42	−0.9
HLA-B*57:01	2	0.56	0.18064	1.36	1.07	−3.39	2.43	−0.95
HLA-B*51:01	2	0.65	0.18064	1.41	1.28	−4.36	2.69	−1.67
HLA-B*07:02	2	0.72	0.18064	1.41	1.19	−3.75	2.6	−1.15
HLA-A*30:01	2	0.73	0.18424	1.41	1.28	−3.73	2.69	−1.05
HLA-A*68:02	2	1.4	0.18064	1.39	0.39	−3.11	1.78	−1.33
HLA-B*44:03	2	1.6	0.18424	1.41	1.28	−4.01	2.69	−1.33
HLA-B*44:02	2	1.9	0.18424	1.41	1.28	−4.16	2.69	−1.47
HLA-A*68:01	2	2.4	0.18064	0.76	0.68	−2.33	1.43	−0.9
HLA-B*40:01	2	3.1	0.18048	1.41	1.28	−4.44	2.69	−1.75

Notes:

1. MHC-I binding score was between 0 and 2. < 0.5 strong binder, 0.5-2 weak binder, > 2 non-binder.

2. The high Immunogenicity score means the degree of the peptide conformity to sequence preference was good.

3. The higher the TAP total score, the higher the likelihood that the peptide will be presented after being swallowed by DCs.

## Discussion

In this study, we have defined and characterized a potential CTL epitope of the spike protein conserved among all the SARS-CoV-2 variants and validated its capacity to elicit IFN-γ and CTL responses of CD8^+^ T cells in the Balb/c mouse model. Furthermore, we found that the epitope, Peptide 2, maybe well recognized by HLA alleles in most populations worldwide.

In recent studies, CD8^+^ T cell immunity was found to make significant contributions to the protective efficacy of SARS-CoV-2 vaccines [24–26]. Additionally, lymphopenia was more accentuated in symptomatic COVID-19 patients with pneumonia than those without pneumonia, consistent with T cell immunity playing a protective role in pre-existing immunity against SARS-CoV-2 [26–28]. However, the role of T cell immunity in the pathology of COVID-19 has not been fully clarified and needs further investigations of T cell epitopes and their functions. Our work has provided a tool to monitor virus-specific CD8^+^ T cells and assess the contribution of CTLs to the control and the elimination of the virus. Based on Pools 2 and 5 of our previous study presented strong T cell stimulation properties [15], we explored those two peptide pools but renamed them as the Pool 1 (S258-512) and Pool 2 (S1015-1275) for further investigation to determine their differences to stimulation of IFN-γ expressions for this study. Other epitopes such as the MHC-I epitope S526-533 (GPKKSTNL) reported from another study [17] were not further investigated since we found that they were much weaker epitopes than Peptide 2 in inducing IFN-γ expressing T cells. A possible explanation for GPKKSTNL might be that the sequence showed a good MHC-I binding only in the H-2D^d^ allele, whereas Peptide 2 showed an excellent binding score in all three alleles.

The SARS-CoV-2 virus was found to mutate rapidly. Accordingly, the development of vaccines protecting people from different virus variants is urgently needed. The neutralizing antibodies induced by vaccines were found to have variable efficacies against the different SARS-CoV-2 variants, and efficacies declined over time, whereas the protection represented by CD8^+^ T cell immunity remained unchanged [24]. Peptide 2 is a highly conserved epitope among all variants and well-presented by MHC-1 of all HLA alleles across the globe. Thus, the conserved Peptide 2 should be suitable for evaluating COVID-19 vaccines for T cell response, particularly for the CD8 T cell-mediated functions. It is also possible to include such an epitope in new COVID-19 vaccines to induce a robust cellular response against all variants of SARS-CoV-2. A recent study confirmed that Peptide 2 probably has a strong cell-mediated immunological function in man; a 9-mer (YLQPRTFLL) peptide overlapped by Peptide 2 could induce a high level of IFN-γ expression from PBMCs of patients who had recovered from COVID-19 and carried the HLA-A*02:01 allele [29]. The 9-mer peptide only showed a relatively good MHC-I binding ability in H-2L^d^ allele (sTable 1), and it stimulated a weaker IFN-γ T cell response than Peptide 2 in mice (sFigure 2).

In conclusion, our study utilized web-based tools to predict human MHC-I epitopes and found several sequences falling into the category. Among these, Peptide 2 (YYVGYLQPRTFLLKY) was not given the most decisive total TAP score in the prediction, but overall it simulated a more robust antigen-specific IFN-γ-expressing CD8+ T response compared to the other predicted epitopic sequences. This epitope sequence is located at the end of NTD of the spike protein and is highly conservative among the currently known SARS-CoV-2 variants and recognizable with the diverse HLA alleles prominent in most world populations. This critical MHC-I epitope can be used to assess CMI induced by COVID-19 vaccines and maybe strategically incorporated into vaccine designs to enhance the prospect of viral elimination by vaccination.

## Supplementary Material

Supplemental Material
