# Supplementary material for: Identification of a promiscuous conserved CTL epitope within the SARS-CoV-2 spike protein
Source: Emerg Microbes Infect. 2022 Mar 1;11(1):730–40. doi: 10.1080/22221751.2022.2043727 (PMC8890520; doi:10.1080/22221751.2022.2043727)
Supplement: Supplemental Material [file TEMI_A_2043727_SM5787.docx]

**Supplementary Data**

**Supplementary Figures**

**sFigure 1.**


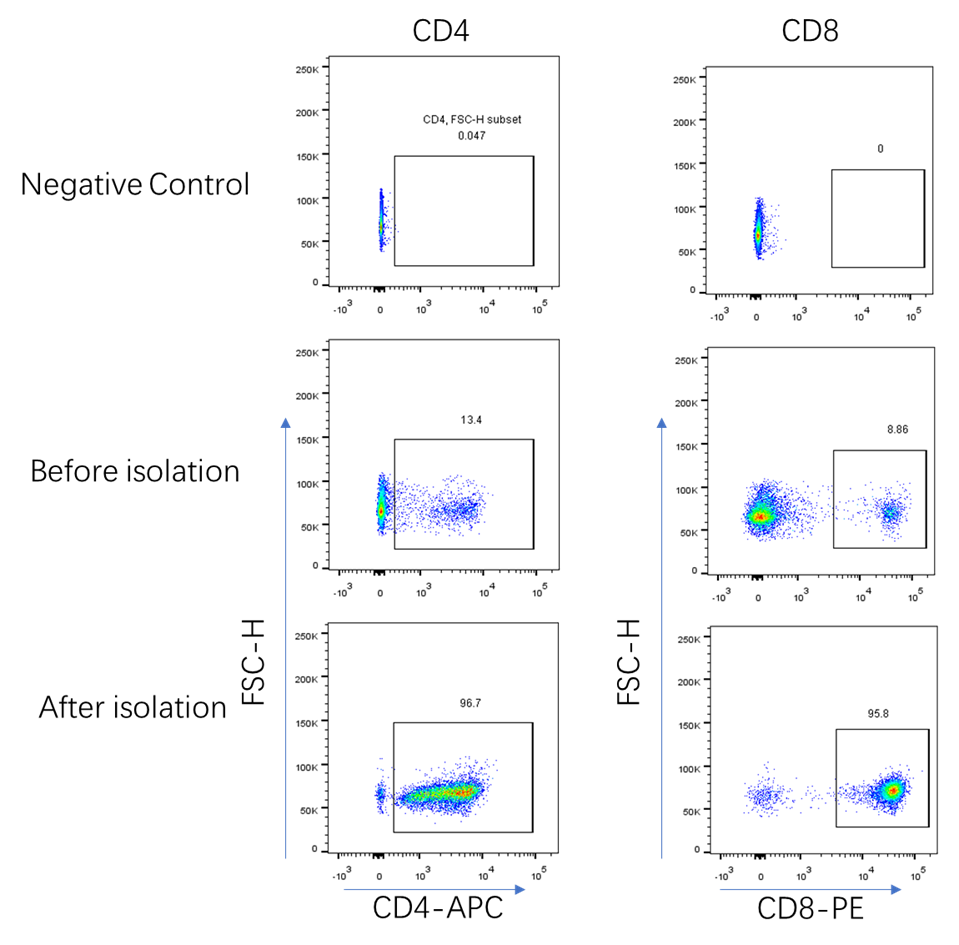


**sFigure1. Cell separation and analysis by flow cytometry.**

Separation of CD4+ T cells and CD8+ T cells from Balb/c mouse spleen.

Splenocytes were collected from mice immunized with pGX9501 and CD4-positive and CD8-positive T cells were separated by magnetic tag. The splenocytes and T cells were stained with CD4-APC and CD8-PE and detected with LSRFortessa flow cytometry (BD) and analyzed by FlowJo (TreeStar). Gates were set on lymphocytes (FSC/SSC).

**sFigure 2.**


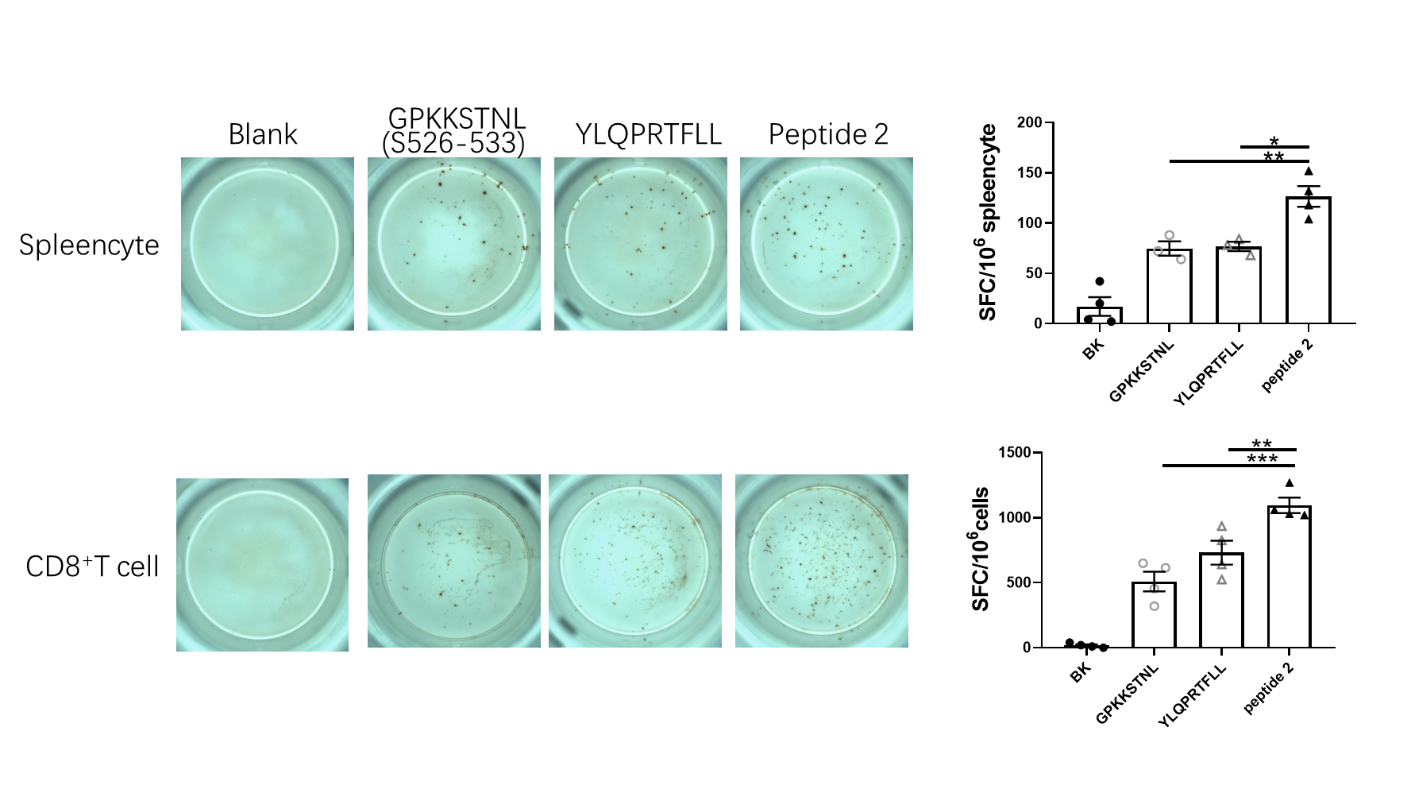


**sFigure2. Peptide 2 induced CD8^+^T cell secreting higher level of IFN-γ.**

BALB/c mice were immunized with the pGX9501. Splenocytes and CD8^+^ T cell were obtained and specific T-cell induction was analyzed with IFN-γ ELIspot, using stimulation with the indicated peptides(S526-533,YLQPRTFLL,peptide 2).

**Supplementary Tables**

sTable 1 MHC-I epitope analysis for Overlapping Peptide 2.

| allele | Peptide number | length | peptide | score |
| --- | --- | --- | --- | --- |
| H-2-Kd | 2 | 8 | GYLQPRTF | 0.62 |
| H-2-Kd | 2 | 9 | GYLQPRTFL | 0.01 |
| H-2-Kd | 2 | 10 | GYLQPRTFLL | 0.1 |
| H-2-Kd | 2 | 10 | VGYLQPRTFL | 0.12 |
| H-2-Kd | 2 | 11 | VGYLQPRTFLL | 1.9 |
| H-2-Kd | 2 | 8 | YLQPRTFL | 0.32 |
| H-2-Kd | 2 | 9 | YLQPRTFLL | 0.98 |
| H-2-Kd | 2 | 11 | YVGYLQPRTFL | 0.1 |
| H-2-Kd | 2 | 9 | YYVGYLQPR | 1.9 |
| H-2-Kd | 2 | 11 | YYVGYLQPRTF | 0.63 |
| H-2-Kd | 2 | 12 | YYVGYLQPRTFL | 0.28 |
| H-2-Kd | 2 | 13 | YYVGYLQPRTFLL | 1.7 |
| H-2-Dd | 2 | 8 | LQPRTFLL | 0.18 |
| H-2-Dd | 2 | 9 | VGYLQPRTF | 0.05 |
| H-2-Dd | 2 | 10 | VGYLQPRTFL | 0.66 |
| H-2-Dd | 2 | 11 | VGYLQPRTFLL | 0.39 |
| H-2-Dd | 2 | 9 | YLQPRTFLL | 0.32 |
| H-2-Dd | 2 | 10 | YVGYLQPRTF | 0.99 |
| H-2-Dd | 2 | 11 | YYVGYLQPRTF | 1.2 |
| H-2-Ld | 2 | 9 | QPRTFLLKY | 0.57 |
| H-2-Ld | 2 | 9 | VGYLQPRTF | 0.54 |
| H-2-Ld | 2 | 10 | VGYLQPRTFL | 1.8 |
| H-2-Ld | 2 | 11 | VGYLQPRTFLL | 1.6 |
| H-2-Ld | 2 | 9 | YLQPRTFLL | 0.56 |
| H-2-Ld | 2 | 10 | YVGYLQPRTF | 1.6 |

**Notes:**

1. MHC-I binding score was between 0 and 2. <0.5 strong binder, 0.5-2 weak binder, >2 non-binder.

**sTable 2. Overlapping Peptide Pool 2.**

| **Peptide number** | **Sequence** | **Start** | **End** | **aa** |
| --- | --- | --- | --- | --- |
| 1 | AAEIRASANLAATKM | 1015 | 1029 | 15 |
| 2 | SANLAATKMSECVLG | 1021 | 1035 | 15 |
| 3 | TKMSECVLGQSKRVD | 1027 | 1041 | 15 |
| 4 | VLGQSKRVDFCGKGY | 1033 | 1047 | 15 |
| 5 | RVDFCGKGYHLMSFP | 1039 | 1053 | 15 |
| 6 | KGYHLMSFPQSAPHG | 1045 | 1059 | 15 |
| 7 | SFPQSAPHGVVFLHV | 1051 | 1065 | 15 |
| 8 | PHGVVFLHVTYVPAQ | 1057 | 1071 | 15 |
| 9 | LHVTYVPAQEKNFTT | 1063 | 1077 | 15 |
| 10 | PAQEKNFTTAPAICH | 1069 | 1083 | 15 |
| 11 | FTTAPAICHDGKAHF | 1075 | 1089 | 15 |
| 12 | ICHDGKAHFPREGVF | 1081 | 1095 | 15 |
| 13 | AHFPREGVFVSNGTH | 1087 | 1101 | 15 |
| 14 | GVFVSNGTHWFVTQR | 1093 | 1107 | 15 |
| 15 | GTHWFVTQRNFYEPQ | 1099 | 1113 | 15 |
| 16 | TQRNFYEPQIITTDN | 1105 | 1119 | 15 |
| 17 | EPQIITTDNTFVSGN | 1111 | 1125 | 15 |
| 18 | TDNTFVSGNCDVVIG | 1117 | 1131 | 15 |
| 19 | SGNCDVVIGIVNNTV | 1123 | 1137 | 15 |
| 20 | VIGIVNNTVYDPLQP | 1129 | 1143 | 15 |
| 21 | NTVYDPLQPELDSFK | 1135 | 1149 | 15 |
| 22 | LQPELDSFKEELDKY | 1141 | 1155 | 15 |
| 23 | SFKEELDKYFKNHTS | 1147 | 1161 | 15 |
| 24 | DKYFKNHTSPDVDLG | 1153 | 1167 | 15 |
| 25 | HTSPDVDLGDISGIN | 1159 | 1173 | 15 |
| 26 | DLGDISGINASVVNI | 1165 | 1179 | 15 |
| 27 | GINASVVNIQKEIDR | 1171 | 1185 | 15 |
| 28 | VNIQKEIDRLNEVAK | 1177 | 1191 | 15 |
| 29 | IDRLNEVAKNLNESL | 1183 | 1197 | 15 |
| 30 | VAKNLNESLIDLQEL | 1189 | 1203 | 15 |
| 31 | ESLIDLQELGKYEQY | 1195 | 1209 | 15 |
| 32 | QELGKYEQYIKWPWY | 1201 | 1215 | 15 |
| 33 | EQYIKWPWYIWLGFI | 1207 | 1221 | 15 |
| 34 | PWYIWLGFIAGLIAI | 1213 | 1227 | 15 |
| 35 | GFIAGLIAIVMVTIM | 1219 | 1233 | 15 |
| 36 | IAIVMVTIMLCCMTS | 1225 | 1239 | 15 |
| 37 | TIMLCCMTSCCSCLK | 1231 | 1245 | 15 |
| 38 | MTSCCSCLKGCCSCG | 1237 | 1251 | 15 |
| 39 | CLKGCCSCGSCCKFD | 1243 | 1257 | 15 |
| 40 | SCGSCCKFDEDDSEP | 1249 | 1263 | 15 |
| 41 | KFDEDDSEPVLKGVK | 1255 | 1269 | 15 |
| 42 | SEPVLKGVKLHYT | 1261 | 1275 | 15 |

**Notes:**

1. The peptides covered the entire sequence of 1015-1275 amino acids in the spike protein as previously named as the Pool 5^15^;
2. The peptides were synthesized with an average length of 15 amino acids and with nine amino acids overlapping each other.

**sTable 3. Prediction scores of peptide 2 and mutated peptide 2**

|  | **Sequence** | **Score** | **MHC-I binding** |
| --- | --- | --- | --- |
| Peptide 2 | YYVGYLQPRTFLLKY | 0.91863 | 0.01 |
| Peptide 2_mut_ | YEVGELQDRTFELKY | 0.025669 | > 2 |

**Notes:**

1. MHC-I binding score was between 0 and 2, where the < 0.5 represents a strong binder, 0.5-2 weak binder, > 2 non-binder.
2. Underlined amino acids were the mutated residues in the Peptide 2_mut_.
